# Temporal Variations and Sociodemographic Differences in no Muscle‐Strengthening Exercise Among Adolescents: A 10‐Year Repeated Cross‐Sectional Study

**DOI:** 10.1111/sms.70233

**Published:** 2026-03-24

**Authors:** Weijun Yu, Raphael H. O. Araujo, Cain Clark, Denver M. Y. Brown, Kai Zhang, Danqing Zhang, Sitong Chen

**Affiliations:** ^1^ School of Teacher Education (School of Physical Education) Taizhou University Zhejiang China; ^2^ Health Research and Innovation Science Centre, Faculty of Health Sciences Klaipeda University Klaipeda Lithuania; ^3^ Faculty of Health, Education and Life Sciences Birmingham City University Birmingham UK; ^4^ School of Health Sciences Kansas State University Manhattan Kansas USA; ^5^ Healthy Active Living and Obesity Research Group Children's Hospital of Eastern Ontario Research Institute Ottawa Ontario Canada; ^6^ School of Human Kinetics University of Ottawa Ottawa Ontario Canada; ^7^ Department of Sports and Health Sciences, Academy of Wellness and Human Development Hong Kong Baptist University Hong Kong China; ^8^ Institute for Health and Sport Victoria University Melbourne Australia

**Keywords:** adolescent, resistance exercise, time trends, youth risk behavioral surveillance system

## Abstract

No or low physical activity (PA) is an emerging risk indicator, but existing studies have not focused specifically on the muscle‐strengthening exercise (MSE) component of the PA guidelines. The aims of this study are to (1) investigate the prevalence of no‐MSE among United States (US) adolescents; (2) examine the sociodemographic correlates of no‐MSE; and finally (3) assess the trends in no‐MSE from 2011 to 2021. Repeated cross‐sectional data from the 2011 to 2021 cycles of the Youth Risk Behavior Surveillance System are used, which include 78 697 US adolescents aged 14–17 years (girls: weighted% = 49.4%). Participants self‐reported their sociodemographic variables and days of MSE. No‐MSE is operationalized as 0 days of MSE per week. Survey‐weighted binary logistic regression models are used to examine the associations between the sociodemographic variables and the prevalence of no‐MSE. Estimates are presented as odds ratios (ORs) and their respective 95% confidence intervals (CI). A total of 28.5% of adolescents reported no‐MSE, of whom girls [OR = 2.12 (2.02–2.23)], older adolescents aged 16 or 17 years (ORs range = 1.31–1.51, *p* < 0.001), Black or African American adolescents [OR = 1.14 (1.05–1.24)], and adolescents with underweight, overweight, or obesity (ORs range = 1.18–1.75, *p* < 0.001) are more likely to report no‐MSE, compared to their respective counterparts. No‐MSE increased from 2011 to 2021 (*p* for trend < 0.001), with the trend seen across all sociodemographic subgroups. Over one in four US adolescents do not engage in MSE, with a higher probability of not engaging among specific demographic and health status‐based subgroups. Given the rising prevalence of no‐MSE and persistent disparities, targeted and tailored interventions as well as equity‐oriented policies are urgently needed to expand access to MSE opportunities for US adolescents.

## 1 Introduction

Historically, physical activity (PA) research has consisted of (1) measuring behavior; (2) characterizing amounts of behavior; (3) examining correlates of behavior; (4) the influence of PA on health; and (5) interventions to promote PA [1]. The links between PA and health outcomes are central to the Physical Activity Epidemiology Research Framework [1, 2], which emphasizes the importance of studying how PA influences health across different populations [3]. As a specific type of PA, muscle‐strengthening exercise (MSE) plays a role in promoting health. MSE includes activities such as using weight machines, exercise bands, hand‐held weights, or body weight exercises like push‐ups and sit‐ups [3]. Building on this, a large body of studies have demonstrated various health benefits of MSE for youth, including improved mental health [4, 5, 6], physical fitness [7], and reduced risk of excess adiposity [4, 8]. This evidence supported the development of the World Health Organization (WHO) PA and Sedentary Behavior Guidelines, which include recommendations specific to MSE [9]. For children and adolescents, these guidelines recommend engaging in MSE three or more times per week [9]. However, adherence to the MSE recommendations remains relatively low and declining [10, 11], with the prevalence being around 19.0% [11].

MSE is an important element in PA and health surveillance [12, 13]. Indeed, there are many ongoing large‐scale surveillance systems that include MSE for children and adolescents. For example, the biggest global surveillance system for school‐based students' health behaviors, the Global School‐based Health Survey [14], which recently has included MSE as a monitoring indicator. In some countries, MSE is also measured by the national health behavior surveillance and monitoring systems, such as the US Youth Risk Behavior Surveillance System (YRBSS) [15] and China's Physical Activity and Fitness in China—The Youth Study [7]. These systems provide population‐based data which can aid in policy development and implementation aimed at improving health‐promoting behaviors. Based on the large‐scale surveillance data aligned to the MSE recommendations, conducting regular MSE surveillance and monitoring is vital to understand levels, patterns, and trends of MSE over time. This information is beneficial for identifying at‐risk population subgroups, developing effective interventions aimed at increasing MSE, and improving PA‐ and health‐related policies for health promotion.

Prior research has demonstrated decreases in the prevalence of meeting the MSE guidelines among US adolescents [16]. Although guideline adherence is a key focus, attention should also be given to those who perform no‐MSE (zero days of MSE). Studies have shown that health benefits can be achieved through any form and amount of PA [17, 18], including minimal or even levels below the recommendations of MSE [19]. In contrast, individuals who engage in no‐MSE may be most vulnerable to health risks [20, 21]. Additionally, no‐MSE is potentially linked to social inequalities or healthcare inequities [22] as those with limited access to resources and opportunities are more likely to be inactive. Therefore, studying no‐MSE could provide additional valuable insights into both health risks and the social disparities in health behaviors. Recently, a new perspective published by Araujo et al. [23] asserted that insights into no or low moderate‐to‐vigorous PA (MVPA) is limited, yet deserving of greater attention as a health risk indicator. To our knowledge, no evidence regarding the prevalence of no‐MSE has been reported among U.S. adolescents. Additionally, previous studies have identified several correlates of adhering to the MSE guidelines [7, 21], among which age, gender/sex, weight status, and race/ethnicity are common sociodemographic factors in adolescents [16]. A point of concern is whether these same correlates are relevant to no‐MSE. Hence, it is important to investigate the prevalence and temporal variations for no‐MSE at the population level, focusing on differences in no‐MSE levels across sociodemographic groups. Therefore, this study aims to (1) investigate the prevalence of no‐MSE; (2) examine differences of no‐MSE across sociodemographic groups; and (3) assess temporal variations of no‐MSE among U.S. adolescents.

## 2 Methods

### 2.1 Study Design and Participants

Data from six cycles of the cross‐sectional YRBSS (2011, 2013, 2015, 2017, 2019, and 2021) are utilized for this study. The YRBSS is conducted biennially to assess health risk behaviors (e.g., PA, smoking, and nutrition) and is designed to yield a nationally representative sample of high school students in the US. It employs a three‐stage cluster sampling method to select students from grades 9 to 12 in both public and private schools [24, 25]. Administered in person by trained research staff, students complete the survey during class time. Each cycle of the YRBSS included in the present study achieves an overall response rate exceeding 60%. The survey results can be weighted to reflect national health data based on the complex survey design. Using these data, a 10‐year independent samples cross‐sectional study is carried out to monitor trends in no‐MSE among US adolescents. The YRBSS survey is approved by the Institutional Review Board (IRB) of the Centers for Disease Control and Prevention. For this secondary analysis, the anonymized data are publicly available; therefore, this study does not require additional IRB approval. Study participants are informed about the survey aims and voluntarily choose to participate, ensuring their privacy is protected. Further details about the YRBSS are available elsewhere [26].

### 2.2 Measures

#### 2.2.1 Sociodemographic

Participants provide sociodemographic information pertaining to their gender (boy/girl), grade (9, 10, 11, and 12), and race/ethnicity (Black/African American, Hispanic/Latino, White, and Other race/ethnicity). Participants self‐report height and weight, from which the body mass index (BMI) is calculated and converted to percentiles using the Centers for Disease Control and Prevention BMI growth charts. Participants are classified into four categories: (1) Underweight, (2) Normal weight, (3) Overweight, and (4) Obesity. Overweight and obesity are determined using the age‐ and gender‐specific ≥ 85th and ≥ 95th percentiles of BMI, respectively [27].

#### 2.2.2 Days of Muscle‐Strengthening Exercise

Participants are asked, “During the past 7 days, on how many days did you do exercises to strengthen or tone your muscles, such as push‐ups, sit‐ups, or weightlifting?” Response options range from 0 to 7 days. Participants reporting 0 days of MSE are regarded as those engaging in “no‐MSE,” adapted from Araujo et al. [23].

### 2.3 Statistical Analysis

All variables included in this study are treated as categorical variables. Missing values for the study variables of interest are addressed by implementing multiple imputations by chained equations to avoid potential biases. We select 20 imputations, following the general rule that the number should be at least as large as the fraction of missing information [28]. For each variable, weighted prevalence estimates with 95% confidence intervals (95% CIs) are calculated, while accounting for complex sampling surveys using Taylor linearization to produce nationally representative prevalence estimates for each survey year. To examine variations in the prevalence of no‐MSE across the 2011 to 2021 rounds of the YRBSS, binary logistic regression models are computed with time‐trend variables across the cycles of data. The survey year is used as a continuous variable to assess whether variation is significant or not. Separate logistic regression models are also computed to explore associations between sociodemographic variables (gender, age, race/ethnicity, and body weight classification) and no‐MSE. Estimates are presented as odds ratios (ORs) with their respective 95% CI. The models are adjusted by gender, age, race/ethnicity, and body weight. All analyses are performed using *survey prefix command* (SVY) procedures, taking sampling stratum, primary sampling unit, and weight based on the YRBSS protocol in Stata/IC 17.0 Basic Edition (Stata Corp LLC). Statistical significance is considered at a 2‐tailed *p*‐value less than 0.05.

## 3 Results

Table 1 presents the characteristics of the 78 697 adolescents aged 14–17 years included in the analysis. Weighted results indicate that the sample is evenly distributed by gender, with 49.4% identifying as girls and 50.6% as boys. The majority of participants are White (53.2%), followed by Hispanic/Latino (23.2%), Black or African American (13.4%), and other ethno‐racial groups (10.3%). Most of the samples (66.4%) are categorized as normal weight, 16.0% as overweight, 14.7% as obese, and 2.9% as underweight. Slightly more than a quarter of adolescents (28.5%) reported no participation in MSE, while 14.6% engaged in daily MSE.

**TABLE 1:** Sample characteristics of this study (weighted results).

Variables	%	95% CI	
Age			
14 years	14.6	13.8	15.5
15 years	28.7	28.2	29.3
16 years	29.1	28.5	29.7
17 years	27.5	26.9	28.1
Gender			
Girl	49.4	48.6	50.3
Boy	50.6	49.7	51.4
Race/ethnicity			
Black or African American	13.4	12.1	14.6
Hispanic/Latino	23.2	21.2	25.1
White	53.2	50.8	55.6
Other race/ethnicity	10.3	9.2	11.3
Body weight			
Underweight	2.9	2.7	3.1
Normal weight	66.4	65.6	67.1
Overweight	16.0	15.6	16.4
Obesity	14.7	14.2	15.3
Days of muscle‐strengthening exercise			
0 day	28.5	27.6	29.4
1 day	8.8	8.5	9.1
2 days	11.3	11.0	11.7
3 days	12.4	12.0	12.9
4 days	8.6	8.2	8.9
5 days	11.3	10.9	11.7
6 days	4.4	4.1	4.6
7 days	14.6	14.1	15.2
Muscle‐strengthening exercise categorization			
Muscle‐strengthening exercise participation	71.5	70.6	72.4
No muscle‐strengthening exercise^a^	28.5	27.6	29.4

Abbreviation: CI: confidence interval.

^a^ Participants reporting 0 days of muscle‐strengthening exercise per week.

Results of the logistic regression analysis are presented in Table 2. Girls have more than twice the odds of reporting no‐MSE compared to boys (OR = 2.12, 95% CI: 2.02–2.23), and older adolescents demonstrate higher odds of no‐MSE compared to younger peers (ORs range from 1.31 to 1.51, *p* < 0.001). Similarly, Black or African American adolescents exhibit a significantly increased likelihood of no‐MSE compared to White adolescents (OR = 1.14, 95% CI: 1.05–1.24), whereas underweight (OR = 1.75, 95% CI: 1.53–2.01), overweight (OR = 1.18, 95% CI: 1.09–1.27), and obese adolescents (OR = 1.68, 95% CI: 1.57–1.80) also show greater odds of reporting no‐MSE compared to their normal‐weight counterparts.

**TABLE 2:** Model of summary on binary logistic regression for correlates of no muscle‐strengthening exercise.

No muscle‐strengthening exercise	OR	95% CI		*p*
Gender (ref = Boy)				
Girl	2.12	2.02	2.23	< 0.001
Age (ref = 14 years)				
15 years	1.06	0.98	1.15	0.126
16 years	1.31	1.20	1.43	< 0.001
17 years	1.51	1.38	1.65	< 0.001
Race/ethnicity (ref = White)				
Black or African American	1.14	1.05	1.24	< 0.001
Hispanic/Latino	0.98	0.91	1.06	0.612
Other race/ethnicity	1.09	1.00	1.20	0.063
Body weight (ref = Normal weight)				
Underweight	1.75	1.53	2.01	< 0.001
Overweight	1.18	1.09	1.27	< 0.001
Obesity	1.68	1.57	1.80	< 0.001

*Note:* Multivariable binary logistic regression model with the Enter (forced‐entry) method; all variables are entered into the model.

Abbreviations: CI: confidence interval; OR, odds ratio; Ref, reference group.

Table 3 displays the trends in no‐MSE among adolescents from 2011 to 2021, stratified by gender. The overall prevalence of no‐MSE increases significantly over time (*p* for trend < 0.001). For the total sample, the ORs for no‐MSE compared to 2011 are 1.16 (2013, 95% CI: 1.03–1.31), 1.07 (2015, 95% CI: 0.95–1.20), 1.12 (2017, 95% CI: 0.98–1.29), 1.17 (2019, 95% CI: 1.04–1.32), and 1.47 (2021, 95% CI: 1.30–1.66), respectively. Among girls, the odds of no‐MSE are significantly higher in 2021 (OR = 1.46, 95% CI: 1.25–1.70), reflecting an upward trend (*p* < 0.001). Boys also exhibit a significant increase in no‐MSE over time, with ORs ranging from 1.22 (95% CI: 1.05–1.41) in 2013 to 1.48 (95% CI: 1.29–1.70) in 2021.

**TABLE 3:** Model summary on results of trend analysis of no muscle‐strengthening exercise in overall and by‐ gender sample.

No muscle‐strengthening exercise	OR	95% CI		*p*	*p for trend*
Overall					
Year (ref = 2011)					
2013	1.16	1.03	1.32	0.019	< 0.001
2015	1.07	0.95	1.20	0.246	
2017	1.12	0.98	1.29	0.100	
2019	1.17	1.04	1.32	0.009	
2021	1.47	1.30	1.66	< 0.001	
Girl					
Year (ref = 2011)					
2013	1.13	0.97	1.32	0.129	< 0.001
2015	1.02	0.89	1.18	0.729	
2017	1.08	0.89	1.31	0.446	
2019	1.11	0.96	1.28	0.165	
2021	1.46	1.25	1.70	< 0.001	
Boy					
Year (ref = 2011)					
2013	1.22	1.05	1.41	0.010	< 0.001
2015	1.14	0.98	1.32	0.091	
2017	1.19	1.03	1.37	0.015	
2019	1.25	1.09	1.44	0.002	
2021	1.48	1.29	1.70	< 0.001	

*Note:* Models are adjusted for age, race/ethnicity, and body weight.

Abbreviations: CI: confidence interval; OR, odds ratio; Ref, reference group.

The trends in no‐MSE from 2011 to 2021 by age group are presented in Table 4. Accordingly, significant upward trends are observed among 14‐year‐olds (*p* for trend = 0.007 < 0.01), 15‐year‐olds (*p* for trend < 0.0001), 16‐year‐olds (*p* for trend < 0.0001), and 17‐year‐olds (*p* for trend < 0.0001). For 15‐year‐olds, the ORs for no‐MSE increase from 1.19 in 2013 (95% CI: 0.98–1.45) to 1.56 in 2021 (95% CI: 1.31–1.86). Similarly, for 16‐ and 17‐year‐olds, no‐MSE prevalence increase significantly, with ORs in 2021 reaching 1.46 (95% CI: 1.26–1.70) and 1.45 (95% CI: 1.30–1.75), respectively.

**TABLE 4:** Model summary on results of trend analysis of no muscle‐strengthening exercise by age.

No muscle‐strengthening exercise	OR	95% CI		*p*	*p for trend*
14 years					
Year (ref = 2011)					
2013	1.11	0.80	1.55	0.531	0.007
2015	0.97	0.69	1.37	0.864	
2017	1.22	0.90	1.66	0.199	
2019	1.27	0.94	1.71	0.118	
2021	1.36	1.03	1.81	0.031	
15 years					
Year (ref = 2011)					
2013	1.19	0.98	1.45	0.079	< 0.0001
2015	1.08	0.92	1.28	0.353	
2017	1.08	0.90	1.30	0.424	
2019	1.18	1.00	1.39	0.056	
2021	1.56	1.31	1.86	< 0.0001	
16 years					
Year (ref = 2011)					
2013	1.22	1.00	1.48	0.052	0.001
2015	1.02	0.87	1.20	0.803	
2017	1.05	0.87	1.28	0.608	
2019	1.12	0.92	1.35	0.256	
2021	1.46	1.26	1.70	< 0.0001	
17 years					
Year (ref = 2011)					
2013	1.12	0.94	1.34	0.215	< 0.0001
2015	1.15	0.96	1.38	0.123	
2017	1.21	1.01	1.44	0.038	
2019	1.18	1.00	1.38	0.047	
2021	1.45	1.20	1.75	< 0.0001	

*Note:* Models are adjusted for gender, race/ethnicity, and body weight.

Abbreviations: CI: confidence interval; OR, odds ratio; Ref, reference group.

Table 5 presents trends in no‐MSE stratified by race/ethnicity. The prevalence of no‐MSE increased over time across all racial/ethnic groups (*p* for trend < 0.01). Hispanic/Latino adolescents demonstrate a significant increase in no‐MSE, as evidenced by an OR of 1.43 in 2021 (95% CI: 1.20–1.70). White adolescents show the most pronounced increase in no‐MSE prevalence, with ORs rising from 1.25 in 2013 (95% CI: 1.06–1.49) to 1.49 in 2021 (95% CI: 1.26–1.76), whereas Black or African American adolescents and those who report other race/ethnicity also demonstrate significant upward trends, although the changes are less pronounced than among White and Hispanic/Latino adolescents.

**TABLE 5:** Model summary on results of trend analysis of no muscle‐strengthening exercise by race/ethnicity.

No muscle‐strengthening exercise	OR	95% CI		*p*	*p for trend*
Black or African American					
Year (ref = 2011)					
2013	1.05	0.86	1.30	0.616	0.007
2015	1.08	0.84	1.39	0.524	
2017	1.08	0.85	1.36	0.520	
2019	1.15	0.90	1.46	0.254	
2021	1.39	1.10	1.75	0.006	
Hispanic/Latino					
Year (ref = 2011)					
2013	1.07	0.89	1.28	0.472	< 0.0001
2015	1.13	0.95	1.34	0.162	
2017	1.12	0.93	1.33	0.231	
2019	1.19	0.96	1.47	0.114	
2021	1.43	1.20	1.70	< 0.0001	
White					
Year (ref = 2011)					
2013	1.25	1.06	1.49	0.010	< 0.0001
2015	1.06	0.89	1.26	0.506	
2017	1.18	0.96	1.45	0.122	
2019	1.16	0.99	1.36	0.072	
2021	1.49	1.26	1.76	< 0.0001	
Other Race/Ethnicity					
Year (ref = 2011)					
2013	1.05	0.82	1.34	0.681	< 0.0001
2015	1.00	0.77	1.31	0.972	
2017	0.98	0.75	1.29	0.891	
2019	1.26	0.98	1.62	0.067	
2021	1.61	1.29	2.01	< 0.0001	

*Note:* Models are adjusted for gender, age, and body weight.

Abbreviations: CI: confidence interval; OR, odds ratio; Ref, reference group.

Table 6 illustrates trends in no‐MSE from 2011 to 2021 by weight status. The prevalence of no‐MSE increases over time among normal‐weight, overweight, and obese adolescents (*p* for trend < 0.01). Obese adolescents demonstrate a significant upward trend, with ORs increasing from 1.12 in 2013 (95% CI: 0.89–1.42) to 1.38 in 2021 (95% CI: 1.11–1.72). Overweight adolescents also show a significant upward trend, with the OR peaking at 1.55 in 2021 (95% CI: 1.25–1.93). Normal‐weight adolescents exhibit a consistent upward trend, with an OR of 1.45 in 2021 (95% CI: 1.25–1.68). For underweight adolescents, the overall trend is not significant, although the 2021 OR reaches 1.63 (95% CI: 1.01–2.61).

**TABLE 6:** Model summary on results of trend analysis of no muscle‐strengthening exercise by body weight.

No muscle‐strengthening exercise	OR	95% CI		*p*	*p for trend*
Underweight					
Year (ref = 2011)					
2013	1.25	0.79	1.98	0.346	0.087
2015	1.54	0.97	2.44	0.066	
2017	1.44	0.85	2.45	0.175	
2019	1.28	0.83	1.98	0.269	
2021	1.63	1.01	2.61	0.044	
Normal weight					
Year (ref = 2011)					
2013	1.16	1.01	1.34	0.041	< 0.0001
2015	1.03	0.90	1.18	0.682	
2017	1.12	0.96	1.32	0.156	
2019	1.13	1.00	1.28	0.052	
2021	1.45	1.25	1.68	< 0.0001	
Overweight					
Year (ref = 2011)					
2013	1.17	0.94	1.46	0.163	< 0.0001
2015	1.18	0.94	1.47	0.151	
2017	1.07	0.87	1.33	0.504	
2019	1.22	0.96	1.54	0.102	
2021	1.55	1.25	1.93	< 0.0001	
Obesity					
Year (ref = 2011)					
2013	1.12	0.89	1.42	0.335	0.003
2015	1.05	0.85	1.29	0.639	
2017	1.10	0.85	1.42	0.464	
2019	1.22	0.97	1.54	0.095	
2021	1.38	1.11	1.72	0.004	

*Note:* Models are adjusted for gender, age, and race/ethnicity.

Abbreviations: CI: confidence interval; OR, odds ratio; Ref, reference group.

## 4 Discussion

To our knowledge, this study is the first to investigate the prevalence and trends of no‐MSE using a nationally representative sample of US adolescents from 2011 to 2021. This 10‐year repeated cross‐sectional study (with independent samples) reveals several key findings. First, the prevalence of no‐MSE is high (28.5%). Second, girls, older adolescents, Black or African American adolescents, and underweight and overweight adolescents are more likely to report no‐MSE compared to their counterparts. Third, a significant upward trend in no‐MSE is observed throughout the study period, with marked increases in 2019 and 2021. Finally, this increasing trend persists across all demographic subgroups.

### 4.1 Findings Interpretations

#### 4.1.1 The Prevalence of No‐MSE

As indicated by a recently published study [23], which suggests considering no or low PA indicators, this study examined the prevalence of no‐MSE in adolescents. A total of 28.5% adolescents reported no‐MSE, which is a relatively high prevalence. As this is one of the very first studies to investigate no‐MSE, little comparable evidence can be taken from the literature, given that most existing studies place more attention on no‐MVPA [23, 29]. Therefore, more studies are needed to investigate no‐MSE among other adolescent populations.

#### 4.1.2 No‐MSE Levels Across Sociodemographic Groups

Our results showed that girls are more likely to report no‐MSE compared to boys. This finding is consistent with those of previous literature on gender/sex related differences in MSE [30, 31], which indicates that boys are more likely to engage in MSE activities than girls [7, 32]. Possible explanations are related to social and gender norms, such as higher parental and school support for boys being involved in PA than for girls [33]. Additionally, adolescent girls frequently demonstrate perceived barriers (e.g., time constraints and body image) [34] in the context of MSE [7], which significantly diminishes their inclination to participate in strength training. Several factors contribute to this, including a lack of confidence, fear of poor performance, and concerns about negative social evaluation [35]. Importantly, these concerns are largely perceptual rather than physiological. When strength training for girls is planned and supervised, it usually improves function and body composition without causing large increases in muscle size [36]. In fact, substantial hypertrophy usually requires high training volumes combined with specific nutritional strategies [37]. Nonetheless, adolescent girls often prioritize achieving a slender body type shape over increasing muscle mass [38], and this misconception may restrict participation in strength training activities.

In addition, we find that older adolescents are more prone to report no‐MSE compared to younger adolescents in this study. This finding is concordant with those of previous studies that have indicated declines in MSE [10] with increasing age during school years. Social and behavioral factors may help explain these results as older adolescents may shift their focus from PA to academic tasks [39, 40], job responsibilities [41], and social interactions [42]. In addition, as adolescents age, they may also spend more time engaging in sedentary behaviors, such as increased screen time [43], and have fewer opportunities for structured physical activity [44]. Declines in organized youth sports and shifting interests (shift toward more recreational and social forms of PA or unstructured activities) [45], along with peer pressure, may further exacerbate the decrease in MSE participation, particularly among older adolescents.

Moreover, in our study, only Black or African American adolescents have significantly higher odds of no‐MSE compared with White adolescents. Interestingly, while our findings diverge from those of previous studies [10, 16], which focused on adherence to MSE guidelines across race/ethnicity groups among US adolescents, these differences are also observed within race/ethnicity groups in Araujo's study related to PA, with socioeconomic status (SES) and access to opportunities for PA practice being key factors behind the inequalities [23]. This is largely due to the fact that White adolescents are more likely to have and maintain healthy patterns compared to their non‐White counterparts [22, 46, 47]. Indeed, race/ethnicity‐related health or behavior disparities have become a concerning public health issue in many culturally diverse countries [22, 46, 47]. More efforts for MSE promotion should be targeted at those who are in minority race/ethnicity subgroups. Sociocultural factors, such as limited access to organized sports, fewer recreational facilities, and economic challenges, particularly for Black and Hispanic/Latino adolescents [48]. Additionally, living in urban or disadvantaged areas further worsens barriers, compounded by the lower socioeconomic status. These disparities reflect systemic racism, residential segregation, and unequal access to resources and health‐promoting environments [22, 49], disproportionately affecting PA opportunities for racial and ethnic minority groups [22].

Furthermore, this study observes a nonlinear (U‐shaped) association between weight status and no MSE. This is consistent with prior evidence that adolescents who perceive themselves as underweight or obese report lower engagement in muscle‐strengthening activities [50], which indicates that barriers are pronounced at both extremes. Both underweight and obesity are associated with higher odds of no‐MSE, whereas overweight shows only a modest elevation. In non–weight‐bearing MSE, adolescents with overweight might demonstrate higher absolute strength than normal‐weight peers, even if relative strength is lower [51]. Among adolescents with obesity, greater mechanical load and reduced movement efficiency can impair performance in strength‐related tasks such as squat jumps and sit‐ups [52], and excess weight might act as a barrier to self‐efficacy and motivation to participate in PA [53]. Additionally, higher body weight is linked to poorer muscular fitness [54]. Positioning MSE as an accessible option for youth with excess weight is a sensible approach and could support healthier body composition and overall well‐being [4]. For underweight adolescents, lower fat‐free mass and absolute strength [55, 56], and in some cases, low energy availability and reduced training tolerance [57] can likewise diminish perceived competence and willingness to participate in MSE. While BMI is widely used as a proxy to assess overweight or obesity, it is highly limited in accurately assessing body composition due to its inability to distinguish between the proportions of fat and lean tissue [58]. Complementing BMI with fat and lean mass estimates and direct strength tests helped distinguish absolute versus relative capacity and accounted for muscle quality changes in obesity [51]. Complementary measures are needed to better assess the association between body composition and health risks [59].

#### 4.1.3 Trend of No‐MSE

A finding of this study is that the prevalence of no‐MSE has increased among US adolescents over the past decade. This important finding can be supported by that of one previous study demonstrating a decreased trend of MSE participation [10, 16] and supported by a large body of PA literature showing a decreased trend of sufficient PA in adolescents [60]. Adolescents are likely to spend a greater amount of time engaged in sedentary activities such as recreational screen time, replacing their opportunities for active time. Additionally, the decreasing trend in daily PE attendance and participation in sports among some U.S. adolescents could further exacerbate reductions in adolescents' opportunities to engage in structured MSE, such as fitness activity during PE lessons [16]. In the present study, the increased trend of no‐MSE remains consistent, irrespective of the subgroup (i.e., gender, age, race/ethnicity, and body weight). This finding suggests that regardless of the sociodemographic characteristics, adolescents tend to engage in no‐MSE. However, more comparable evidence to support or negate the current study is needed. Future research is encouraged to further assess and validate the trends of no‐MSE engagement in adolescents. Another key observation is the significant increase in the likelihood of no‐MSE across all groups in 2021. This trend is likely compounded by the COVID‐19 pandemic, which restricted access to traditional PA facilities, like gyms, thereby reducing participation in MSE [61]. This finding highlights the importance of creating flexible and scalable programs, such as at‐home or online MSE training programs, to keep adolescents engaged in MSE during periods of isolation. Beyond the pandemic contexts, adding equipment‐based MSE to PE classes and offering home routines through school and community programs can reduce barriers and decrease the prevalence of no MSE. Furthermore, school closures, social isolation, and mental health issues during the pandemic may have reduced adolescents' MSE participation. The disparities observed in MSE trends are likely influenced by a complex mix of biological, environmental, cultural, and geographic factors, all of which shape how adolescents engage in physical activities and develop muscular strength. More research is needed to explore these trends, including time‐trend studies to assess whether these rates recover post‐pandemic, as well as to evaluate effective interventions to combat the rise in no‐MSE and examine the effectiveness of strategies aimed at reducing no‐MSE.

### 4.2 Strengths and Limitations

To our knowledge, this is the first study to examine trends in no‐MSE among adolescents over a decade, with further stratification by gender, age group, race/ethnicity, and body weight categorization. The strength of this study lies in its use of data from a nationally representative sample of 78 697 US adolescents. Nevertheless, limitations include the reliance on self‐reported questionnaires for measuring MSE, which may introduce recall and social desirability biases. However, current device‐measured PA data are unable to capture domain‐specific PA (e.g., aerobic or MSE). This study includes six YRBSS waves to describe trends. However, each wave is cross‐sectional with independent samples; therefore, longitudinal designs are needed to test causal pathways. While this study provides stratified findings by gender, age, and race/ethnicity, it does not include other sociodemographic variables known to influence movement behaviors, such as household income and parental education, or explore the interactions among these variables. Future studies should estimate joint effects on no‐MSE across combinations of risk factors and assess interactions.

### 4.3 Implications

These findings highlight a concerning rise in the prevalence of no‐MSE, particularly among certain demographic subgroups, emphasizing the need for targeted interventions to address this growing public health issue. Given the relatively high level of no‐MSE (28.5%), addressing this is critical for adolescent health. While meeting the MSE guidelines is ideal, existing evidence suggests that even minimal engagement in MSE, such as 1 day per week, provides notable health benefits compared to no engagement at all [3, 62]. Therefore, reducing the prevalence of no‐MSE should be a key goal of adolescent health promotion. Interventions should focus on shifting adolescents from “doing nothing” to “doing something,” promoting even small amounts of MSE as a meaningful step. Moreover, increasing MSE participation is essential for adolescent health. Integrating strength‐based activities in schools and improving community access to exercise facilities are effective strategies to promote MSE participation among youth.

## 5 Conclusion

The prevalence of no‐MSE is relatively high and has consistently increased among US adolescents from 2011 to 2021. Notably, girls, older adolescents, Black or African American adolescents, and those with excess weight present a higher prevalence of no‐MSE. Monitoring of no‐MSE could help provide important information on those least active populations that can be targeted via interventions. Given that the most substantial benefits of increased MSE are accrued by the least active, targeted interventions and policy initiatives are crucial. These efforts are essential not only to reduce the prevalence of no MSE but also to address disparities related to gender, age, and ethnicity among US adolescents, potentially yielding substantial and economical health benefits.

## 6 Perspective

This study indicates a rising prevalence of no‐MSE with disparities concentrated in girls, older adolescents, and racial/ethnic minorities. To address these inequities, future research should focus on developing effective, context‐sensitive strategies. Future studies could extend our findings by examining how multiple intersecting risk factors (e.g., adolescent girls at older ages) jointly contribute to no‐MSE as many adolescents experience these risk factors simultaneously. Moreover, studies should prioritize longitudinal cohorts and randomized trials to establish causality and dose–response in these populations. Additionally, there is a need for more internationally comparable data on MSE to advance this research field.

## Funding

The authors have nothing to report.

## Conflicts of Interest

The authors declare no conflicts of interest.

## Data Availability

The data that supports the findings of this study are available in the Supporting Information of this article.
